# Supplementary material for: Selective Blockade of Two Aquaporin Channels, AQP3 and AQP9, Impairs Human Leukocyte Migration
Source: Cells. 2025 Jun 11;14(12):880. doi: 10.3390/cells14120880 (PMC12191162; doi:10.3390/cells14120880)
Supplement: Supplementary file 1 [file cells-14-00880-s001.zip › Supplementary material-Figure S1.pdf]

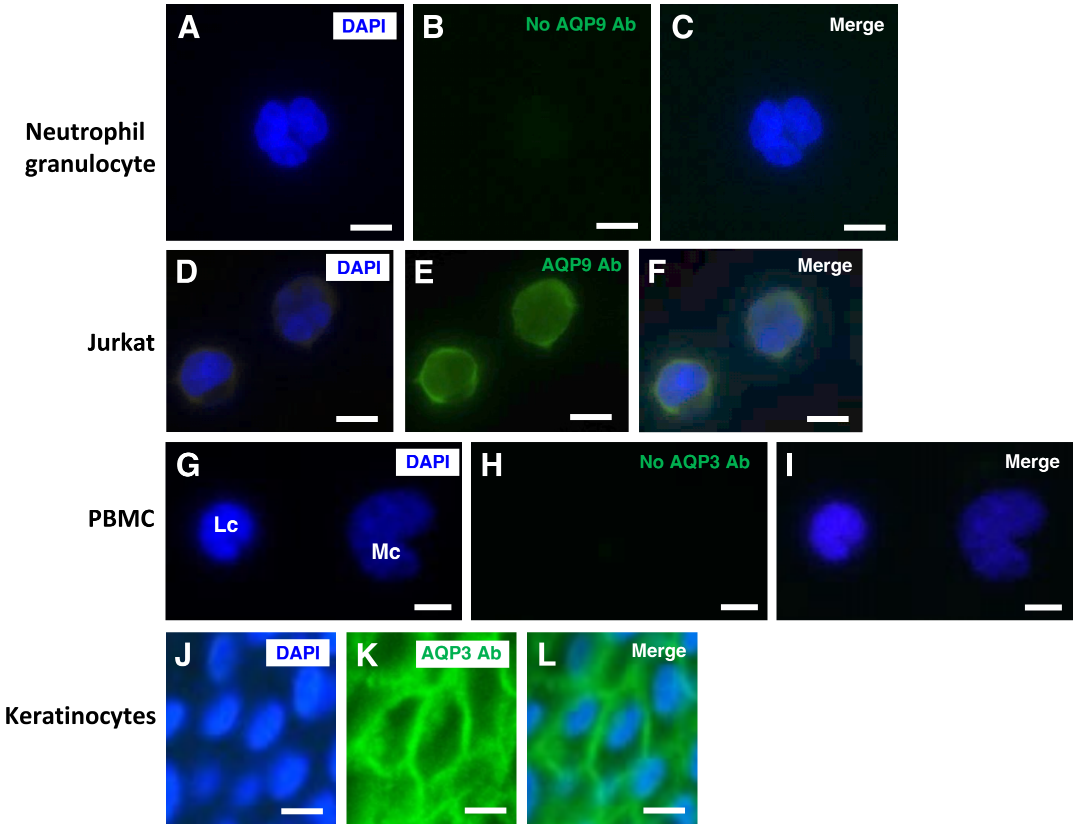

Figure 1sm. Controls for primary and secondary antibody fluorescence. (**A, B, C**) Anti-AQP9 antibody negative control. No fluorescence is seen when isolated human neutrophil granulocytes are incubated with the secondary antibody by omitting the AQP9 antibody (**B**, **C**). (**D**, **E**, **F**) Anti-AQP9 antibody positive control. Immunofluorescence is seen at the plasma membrane of Jurkat cells (**E**, **F**; green fluorescence). (**G**, **H**, **I**) Anti-AQP3 antibody negative control. No fluorescence is seen when isolated human PBMC are incubated with the secondary antibody by omitting the AQP3 antibody (**H**, **I**). (**J, K**, **L**) Anti-AQP3 antibody positive control. Immunofluorescence is seen at the plasma membrane of human keratinocytes (**K**, **L**; green fluorescence). Cell nuclei are counterstained with DAPI fluorophor (**A**, **C**, **D**, **F**, **G**, **I**, **J**, **L**; blue fluorescence). Scale bars, 10 µm.
