# Supplementary material for: Selective Blockade of Two Aquaporin Channels, AQP3 and AQP9, Impairs Human Leukocyte Migration
Source: Cells. 2025 Jun 11;14(12):880. doi: 10.3390/cells14120880 (PMC12191162; doi:10.3390/cells14120880)
Supplement: Supplementary file 1 [file cells-14-00880-s001.zip › Supplementary material-Table S1.pdf]

cells

MDPI

Table 1. Forward and reverse primer sequences for Real time quantitative PCR.

Primer		Sequence	PCR Product length	Reference
AQP1	Forward	5′-GTAGCCAGCACGCATAGCAC-3′	140 bp	[20]
	Reverse	5′-GCCATCCTCTCAGGCATCAC-3′		
AQP3	Forward	5′-GGAATAGTTTTTGGGCTGTA-3′	159 bp	[20]
	Reverse	5′-GGCTGTGCCTATGAACTGGT-3′		
AQP5	Forward	5′-TCCATTGGCCTGTCTGTCAC-3′	134 bp	[20]
	Reverse	5′-ACCCAGAAAACCCAGTGAGC-3′		
AQP9	Forward	5′-ATGTGGGAGCCCAGTTCTTG-3′	151 bp	[20]
	Reverse	5′-TACGGAGCTGGGTATGTTGC-3′		
β-actin	Forward	5′-CTGTGGCATCCACGAAAC-3′	88 bp	[55]
	Reverse	5′-CAGACAGCACTGTGTTGG-3′		

20 Thon, P.; Rahmel, T.; Ziehe, D.; Palmowski, L.; Marko, B.; Nowak, H.; Wolf, A.; Witowski, A.; Orlowski, J.; Ellger, B.; et al. AQP3 and AQP9-Contrary Players in Sepsis? *Int J Mol Sci* **2024**, *25*, doi:10.3390/ijms25021209.

55 Taihi, I.; Nassif, A.; Berbar, T.; Isaac, J.; Berdal, A.; Gogly, B.; Fournier, B.P. Validation of Housekeeping Genes to Study Human Gingival Stem Cells and Their In Vitro Osteogenic Differentiation Using Real-Time RT-qPCR. *Stem Cells Int* **2016**, *2016*, 6261490, doi:10.1155/2016/6261490.
